# Future of Colorectal Cancer Screening: From One-Size-FITs-All to Tailor-Made

**DOI:** 10.3389/fgstr.2022.906052

**Published:** 2022-06-23

**Authors:** Tim Kortlever, Manon van der Vlugt, Evelien Dekker

**Affiliations:** Department of Gastroenterology and Hepatology, Amsterdam University Medical Centers, University of Amsterdam, Amsterdam, Netherlands

**Keywords:** colorectal cancer, screening, risk, prediction model, fecal test, blood test, risk factors

## Abstract

Screening for colorectal cancer (CRC) and its precursor lesions, advanced adenomas (AA), has been shown to effectively reduce CRC-related mortality. However, the method of CRC screening varies among countries. Primary colonoscopy screening is the most effective screening option from an individual point of view, but it is costly and population-wide participation rates are relatively low. Repeated screening with a fecal immunochemical test (FIT) is a non-invasive and inexpensive way to select individuals at high risk for CRC for colonoscopy. Despite its widespread use and mostly high participation rates, FIT is not perfect. Its sensitivity for advanced neoplasia (AN) is low. Besides, the false positivity rate of FIT is relatively high. This leads to unnecessary colonoscopies, anxiety, and risks among FIT-positives. New strategies need to be developed to improve CRC screening. In the past years, much research has been undertaken on risk-based screening or risk models. These include tests consisting of multiple risk factors and/or biomarkers that either assess the risk of disease at a single point in time (cross-sectional risk models) or predict the risk of developing CRC in the future (longitudinal risk models). We provide an overview of the developments on risk models for CRC screening and discuss some of the obstacles that need to be overcome to enable widespread implementation in existing CRC screening programs.

## Introduction

Colorectal cancer (CRC) is the third most common cancer and the second most lethal cancer in the world (1). In 2020, around 2 million individuals were diagnosed with CRC. Patients often remain asymptomatic until the tumor is already in an advanced stage, which partly explains why around 50% of patients die from CRC. Screening aims to reduce CRC-related mortality in two ways (2): First, it aims at detecting tumors at an earlier stage, which increases the chance of curative treatment (3). Second, screening intends to find relevant precursor lesions, such as advanced adenomas and advanced serrated polyps, which can be removed at colonoscopy before they develop into CRC. 

The foundations for CRC screening were laid in the 1970s, when advances in colonoscopy made it possible to remove polyps (4). The guaiac fecal occult blood test (gFOBT) was developed around the same time, which provided an important tool for detecting individuals who likely had polyps or CRC. Soon thereafter the first randomized controlled trials were initiated, demonstrating that screening by gFOBT followed by colonoscopy led to a reduction in CRC-related mortality (5–7).

Today, many developed countries have CRC screening programs either as an opportunistic program or an organized program. Countries that apply an opportunistic approach usually offer a range of screening tests to the population, mostly colonoscopy, CT-colonography or the fecal immunochemical test (FIT) – the successor of the gFOBT. Organized screening programs on the other hand actively and repeatedly invite their target population for screening. Most use a non-invasive test such as FIT to select high-risk individuals for follow-up colonoscopy.

However, current screening tests are not optimal. Although colonoscopy is the best method to find cancer and to detect and remove precursor lesions it is an invasive and costly procedure. These costs may not always be fully reimbursed, which particularly affects individuals with low income who may have a higher risk of CRC. In contrast, FIT is less sensitive for AN, but it is more user-friendly than colonoscopy and it has higher participation rates (8, 9). FIT may also be more cost-effective than colonoscopy (10). Another downside of existing screening tests, is that screening programs generally apply a uniform starting age and test positivity threshold. For example, individuals in the Netherlands all receive an invitation for screening with FIT at a cut-off of 47 µg Hb/g feces from age 55 despite the fact that not all individuals all have the same risk of having or developing CRC. An ideal screening strategy would use a test that is more sensitive than FIT (without being less specific, i.e. raising the number of individuals selected for colonoscopy), at least as user-friendly as FIT, cost-effective, and adaptable to different risk groups.

**Figure 1:**
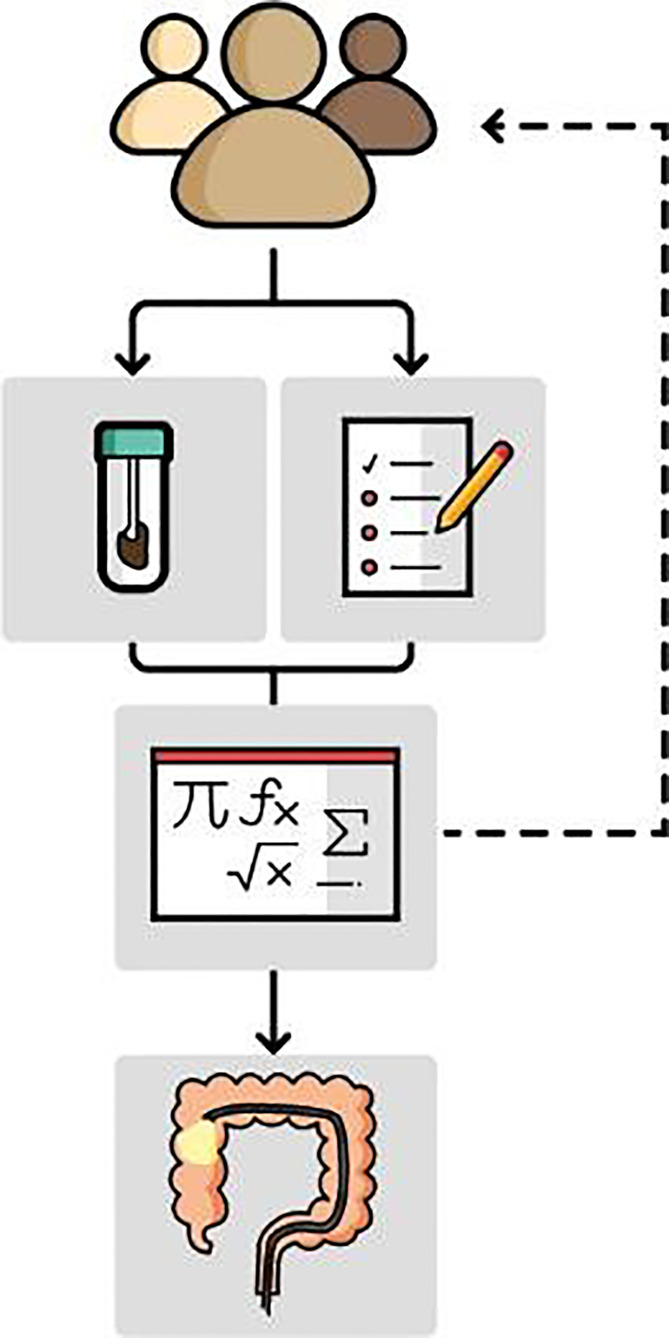
Cross-sectional risk models may be used in a similar way as the FIT is currently used. Risk models estimate the risk of detection of AN at colonoscopy based on multiple variables, such as risk factors collected in a questionnaire or patient records, and fecal biomarkers. Those with an elevated risk are invited for follow-up colonoscopy. Individuals with a low risk are re-invited in a following screening round.

Risk-based screening might be a way to improve CRC screening. It involves the combination of several risk factors and/or biomarkers into a risk model, risk score, or another type of algorithm. Risk-based screening can be roughly divided into two types: cross-sectional and longitudinal risk-based screening. In cross-sectional screening, risk factors and/or biomarkers are combined to assess the risk of having AN at the moment the test is used. These tests, which we will refer to as risk models, can be used to select individuals for colonoscopy, comparable to how current FIT-based screening programs operate (**Figure 1**). In longitudinal risk-based screening, the combination of risk factors and/or biomarkers is used to assess the risk of developing AN in the future, for example within 10 years. These risk models can be used to inform participants of their long-term risk and offer different screening strategies according to their risk.

In this review, we will discuss the current evidence for markers and risk models for cross-sectional or longitudinal use in CRC screening. We will also discuss the hurdles that need to be overcome for successful implementation in established screening programs.

## Cross-Sectional Risk-Based Screening

### Risk Models Using Demographic and Habitual Risk Factors

The first approach in risk-based screening is to combine demographic and/or clinical risk factors that are associated with CRC or AN. Examples include age, sex, BMI, family history, alcohol consumption, smoking habits, diet, and medication use. The advantage of these risk factors is that most can be measured relatively easy: data can either be collected from existing databases or patient records, or they can be obtained through a questionnaire.

The Asia-Pacific Colorectal Screening (APCS) score is a prime example (11). This score was derived with a multivariable logistic regression analysis in 860 asymptomatic subjects undergoing a screening colonoscopy. The APCS assigns points based on age, sex, family history of CRC in a first-degree relative, and smoking behavior. Its performance was measured with the c-statistic, a measure that indicates the ability of a risk model to predict the outcome. A c-statistic of 0.5 means that the model is not better than flipping a coin, while a c-statistic of 1 means that the model can perfectly discriminate between those with and without the outcome. The c-statistic of the APCS for detecting AN was 0.64 in a validation cohort consisting of asymptomatic individuals (**Table 1**). Discrimination might be improved by adding BMI into the APCS score (12), although another study from Kaminski et al. suggests there is no added benefit of this variable (13). Several other groups have also included dietary information, such as the consumption of vegetables and red meat, in large cohorts to various degrees of success: Cai et al.’s model had the best performance (c-statistic: 0.74) using the variables age, sex, smoking, diabetes, and consumption of green vegetables, pickled food, fried food, and white meat (14). Two other models that included dietary information performed less well (15, 16).

**Table 1:** Performance of a selection of the reported risk models that use demographic and habitual risk factors, including combinations with FIT.

Study	Risk model	Target condition	C-statistic (95% CI)	Sensitivity (%)	Specificity (%)
Yeoh et al. (11)	Age, sex, family history, smoking (APCS)	AN	0.64 (0.60 – 0.68)	-	-
Kim et al. (12)	Age, sex, BMI, smoking, family history of CRC	AN	0.68	85.7	33.4
Kaminski et al. (13)	Age, sex, BMI, smoking, family history of CRC	AN	0.62 (0.60 – 0.64)	-	-
Cai et al. (14)	Age, sex, smoking, diabetes mellitus, green vegetables, pickled food, fried food, white meat consumption	AN	0.74 (0.70 – 0.78)	–	–
Lin et al. (15)	Age, previous sigmoidoscopy/colonoscopy and detection of polyps, family history of CRC, smoking, physical activity, vegetable consumption, BMI, NSAID use, estrogen use (women only)	AN	0.62 (0.58 – 0.65)	-	-
Tao et al. (16)	Sex, age, first-degree relative with history of CRC, smoking, alcohol consumption, red meat consumption, NSAID use, previous colonoscopy, previous detection of polyps	AN	0.66	–	–
Aniwan et al. (17)	FIT (≥50 ng/ml) and APCS (age, sex, family history, smoking) ≥4	AN	0.67	29.2	95.5
Chiu et al. (18)	APCS (age, sex, family history, smoking) ≥4 or FIT (20 μg Hb/g)	AN	–	70.6	–
Park et al. (19)	Age, smoking, diabetes mellitus, square root of FIT	AN	0.75 (0.73 – 0.78)	-	-
Soonklang et al. (20)	Age, BMI, alcohol consumption, smoking, FIT	AN	0.77 (0.71 – 0.84)	–	–
He et al. (21)	FIT, age, BMI, family history of CRC, diabetes, smoking, alcohol consumption	AN	0.69 (0.65 – 0.73)	76.7	-
Kortlever et al. (22)	FIT, square root of FIT, age, sex	AN	0.71 (0.65 – 0.78)	28.7	96.9
Cooper et al. (23)	FIT, age, sex, previous screening history	AN	0.69 (0.66 – 0.71)	33.2	84.7
Stegeman et al. (24)	FIT, age, calcium intake, smoking, family history of CRC	AN	0.76	40.6	94.0

Aiming at better performance, the abovementioned risk factors can also be combined with FIT. One can argue that this makes it more difficult to collect all information for risk calculation, because this necessitates the collection of feces. However, adding these risk factors to FIT can be relatively simple in screening programs that already use FIT. Risk factors and FIT can be used sequentially or combined into a single risk model. In studies investigating the former, a risk score based on several risk factors was used to distribute asymptomatic individuals into low-, medium-, and high-risk groups. Colonoscopy was offered to individuals with a high risk according to the risk score, while those with a low to medium risk were offered a FIT with subsequent colonoscopy if positive (17, 18, 25). This strategy is currently being compared to one-time colonoscopy and annual FIT in the randomized TARGET-C trial (26). In the interim-analysis of this trial, the yield of risk-based screening was significantly better than FIT (OR 1.49, 95%CI: 1.13 – 1.97), although relatively more individuals were invited for colonoscopy than in the FIT only arm. While this approach may be a viable solution for opportunistic screening programs looking to reduce the number of colonoscopies, it may not be feasible for population-wide screening programs that operate with limited colonoscopy capacity.

Instead of sequential screening, FIT and other risk factors can also be integrated into a risk model. Such models calculate the risk of AN given the presence or absence of risk factors, including for example the quantitative result of FIT. As risk can be expressed as a number between 0 and 1, a cut-off point can be selected to determine which individuals are at highest risk of AN and are advised a follow-up colonoscopy. Indirectly, this leads to individualized FIT cut-offs: for example, a 70-year old male, who smokes, and has a high BMI may need a lower FIT result to cross the risk threshold, since his risk is already high, compared to a 55-year old female with a normal BMI who does not smoke. One example is the model published by Stegeman et al., which combined FIT with age, calcium intake, smoking, and family history of CRC in a sample of 1,112 asymptomatic individuals. This risk model significantly improved detection of AN compared to FIT only, without increasing the number of colonoscopies (24). Similar improvements were seen in other models: Park et al. reported their model, which consisted of FIT, age, smoking, and diabetes, with a c-statistic of 0.75 compared to 0.68 with FIT only (19). Soonklang et al. and He et al. also observed improvements in the c-statistic when combining their selected risk factors with FIT (21).

A disadvantage of using habitual risk factors is that a questionnaire might be needed to gather risk factor information, which may affect participation rates. Results from the TARGET-C trial indeed demonstrate this issue: 94% of study participants underwent screening in the FIT screening group compared to 85% in the risk-based screening group (18, 26). Therefore, models that combine readily available information with FIT may be more practical. For example, demographic risk factors such as age and sex are almost always readily available. Studies in symptomatic individuals referred for colonoscopy suggest that a simple model with FIT, age, and sex may improve yield of AN compared to FIT-only (27, 28). However, this was not confirmed in a cohort of average-risk individuals (22). Information from previous screening rounds may also be useful. Cooper et al. published a model that combined FIT with age, sex, and participation status in the previous screening round (23). This model had a higher c-statistic than FIT only. Data also suggest that the quantitative FIT result of previous screening rounds may be associated with detection of AN in later rounds, which could be useful in future risk models (29, 30).

### Fecal-Based Markers and Risk Models

Developing new fecal biomarkers may also improve the yield of AN compared to FIT-only. The best-known example is the multitarget stool DNA (mt-sDNA) test, also known as Cologuard™ (Exact Sciences, Madison, WI). The mt-sDNA is a model consisting of quantitative assay results of FIT and several genetic markers found in stool samples, namely *KRAS* mutations, aberrant *NDRG4* and *BMP3* methylation, and a marker for total human DNA, *β-actin* (**Table 2**) (31). In their validation study in 9,989 participants who underwent screening colonoscopy, Imperiale et al. reported that the mt-sDNA test had superior sensitivity for CRC, advanced adenomas and sessile serrated polyps of 10mm or larger. However, this came at the expense of a significantly higher false positivity rate and thus more unnecessary colonoscopies (32). In addition, this version of the mt-sDNA test requires participants to collect a whole stool sample, which is less user-friendly than tests only requiring a small sample (e.g. FIT). Modelling studies have suggested that triennial mt-sDNA is less cost-effective than biennial FIT at current reimbursement rates, which is a concern for population-wide screening programs (38, 39). An updated version of the mt-sDNA test is currently being evaluated in 24,000 individuals undergoing a screening colonoscopy (40). Other models using DNA markers have been reported, albeit in smaller study groups and without external validation (41–43). Individual markers are also being explored, such as *SDC2* (33). This marker is currently under investigation in a large prospective trial in asymptomatic individuals undergoing colonoscopy screening (44).

**Table 2:** Performance of a selection of the reported risk models that use faecal biomarkers.

Study	Risk model	Target condition	C-statistic (95% CI)	Sensitivity (%)	Specificity (%)
Imperiale et al. (31)	KRAS, NDRG4, BMP3, β-actin, Hb	AN	0.73	46.4	86.6
Bosch et al. (32)	KRAS, NDRG4, BMP3, FIT	AA/ASP	–	46.0	89.0
Wang et al. (33)	SDC2	AN	-	79.9	98.0
Kim et al. (34)	Calgranulin B, FIT, age	CRC	0.93	79.8	90.0
De Klaver et al. (35)	Hb, calprotectin, serpin family F2	AN	-	42.9	96.6
Wong et al. (36)	Fusobacterium nucleatum, FIT	CRC	0.96	82.6	94.8
Bosch et al. (37)	VOCs	CRC	0.96	100	100
		AA	0.96	96.9	93.8

Calprotectin is another potential marker for AN that has been frequently discussed (45–49). Although calprotectin by itself seems not useful for screening purposes, promising results have been achieved by combining FIT with calprotectin. In one study, researchers observed a significantly improved c-statistic when calgranulin B, part of calprotectin, was added in a model to FIT and age (34). Recently, De Klaver et al. published a study in which they tested a CART model consisting of assays for hemoglobin, calprotectin, and serpin family F member 2 (dubbed the multitarget FIT or mtFIT) (35). In a cohort of 1,284 individuals, the majority of whom were asymptomatic individuals undergoing screening colonoscopy, the mtFIT had a sensitivity for AN of 42.9%, compared to 37.3% for FIT only (*p* = 0.03). Results of an early health technology assessment, reported in the same paper, indicated that mtFIT would be more cost-effective than FIT in a population-wide screening program when its price would stay below €59 per test.

Interest has also grown in the gut microbiome. Data suggests that particular bacteria or species, or dysbiosis of gut microbiota, are related to colorectal carcinogenesis or that they might be associated with the presence of AN (50, 51). Regardless of whether gut microbiome profiles represent the chicken or the egg, more than a dozen studies have identified markers or profiles that can be used for screening. Most are case-control studies with limited size (51). An example is the study by Wong et al., which found that the combination of FIT and *Fusobacterium nucleatum* (*Fn*) yielded a significantly better sensitivity for CRC and AA than FIT only (36). Currently, a large Norwegian trial is underway with the aim of developing an algorithm to identify microbiome profiles in FIT-positive individuals (52). This study will also investigate the role of diet, lifestyle, and prescription drugs in the relation between gut microbiome and CRC.

Future risk models may also include Volatile Organic Compounds (VOC). VOCs are chemicals released as gases from liquids or solids, for example stool, urine, or the air we exhale. Cancer may induce different patterns in metabolic substrates, which can be detected by an e-nose or techniques such as gas chromatography mass spectrometry. Limited evidence for fecal VOCs is available, consisting of only case-control studies (53). One of the largest studies was conducted by Bosch et al., who observed very good discrimination (37). Besides stool, researchers are also using breath and urine to find VOC patterns for CRC screening (54).

### Blood-Based Markers and Risk Models

Risk models that use markers from peripheral blood, sometimes called liquid biopsies, are also suggested for screening. Similar to stool-based markers, several types of biomarkers can be distinguished: messenger RNA (mRNA), microRNA (miRNA), cell-free DNA (cfDNA), circulating tumor DNA (ctDNA), proteins, and extracellular vesicles (EV). Multiple (systematic) reviews have provided a comprehensive overview of this rapidly expanding field (55–59).

One of the most studied markers from peripheral blood is circulating methylated SEPT9 (mSEPT9). This marker is FDA-approved and available under the name EpiproColon (Epigenomics AG). In 2013, Church et al. published a large nested case-control study in which they examined this ctDNA marker in 1,510 asymptomatic individuals undergoing screening colonoscopy (**Table 3**) (60). Since then, a new version of the mSEPT9 test was developed with potentially increased performance (61, 65). A large clinical trial is currently in progress to investigate the clinical utility of repeated mSEPT9 testing in an average risk population (66).

**Table 3:** Performance of a selection of the reported risk models that use blood-based biomarkers.

Study	Risk model	Target condition	C-statistic (95% CI)	Sensitivity (%)	Specificity (%)
Church et al. (60)	Methylated SEPT9	CRC	-	48.2	91.5
Johnson et al. (61)	Methylated SEPT9	CRC	–	73.3	81.5
Wan et al. (62)	Cell-free DNA	Early-stage CRC	0.92	85.0	85.0
Kleif et al. (63)	Age, sex, CEA, hsCRP, HE4, ferritin	CRC	0.70 (0.66 – 0.74)	18.0	90.0
Otero-Estévez et al. (64)	Soluble CD26, FIT	AN	-	56.1	93.5

Other genetic markers extracted from peripheral blood are less well studied, but harbor potential. For example, Wan et al. developed a panel of cfDNA markers using artificial intelligence in a case-control study (n= 817) and found a mean c-statistic of 0.92 (62). Validation of this panel is currently underway in a trial with an estimated sample size of 25,000 individuals (67).

Protein markers could also be useful for screening. A Danish group has gathered several proteins associated with CRC, including the well-known carcinoembryonic antigen (CEA) (68–70). While their first studies were performed in cohorts of patients or symptomatic individuals, they recently published a large development and validation study of an expanded model (63). Despite promising results achieved earlier, the c-statistic of the model was only 0.61 for detecting AN and the sensitivity and specificity were inferior to those of FIT. Another group has looked at soluble CD26 (sCD26) and found a c-statistic of 0.75 in a group of 516 asymptomatic individuals with a first-degree relative with CRC, compared to a c-statistic of 0.72 of FIT (64). Combination of sCD26 and FIT yielded better performance than either sCD26 or FIT separately. Such combinations or using a blood-based test as an add-on test after FIT, may be useful for reducing the number of false positives (71).

## Longitudinal Risk-Based Screening

The lifetime risk of developing CRC is 4-5% (72). Identifying those who have an increased risk of developing CRC in the future as well as those who have a negligible risk might be useful for developing personalized screening strategies. For example, those at high risk could be screened more frequently (e.g. annual FIT instead of biennial) or start screening earlier and vice versa for those at a low risk. In theory, this would provide optimal prevention for high-risk individuals, while those with a negligible risk are protected against the potential adverse effects of screening. Longitudinal risk models are developed with the aim of identifying these different groups.

An example is the QCancer risk calculator (73). Based on demographic factors (age, sex, ethnicity), habitual risk factors (BMI, smoking status), medical history, and familial history of cancer, one can estimate the absolute risk of developing CRC and seven other types of cancer within the next 10 years. In an external validation study of 11 longitudinal prediction models for CRC, this model reached a c-statistic of 0.70 in men and 0.66 in women, better than any other model in the study (74). Subsequently, this model was used by an expert panel to provide screening recommendations based on the long-term CRC risk and the simulated benefit of available screening options (colonoscopy, sigmoidoscopy, biennial FIT) (75). They suggested that those with a predicted 15-year CRC risk of 3% or lower may have limited benefits from screening and could choose to abstain from participation.

Long-term predictions can also be made using genetic information. Genome-wide association studies (GWAS) have suggested that specific SNPs may be associated with CRC (76, 77). Numerous studies using SNPs in a polygenic risk score (PRS) or genetic risk score (GRS) have since been published. A systematic review by McGleogh et al. found that most had poor discrimination (78). Similar to cross-sectional models, genetic information can be combined with information on habitual or demographic risk factors. For example, Jeon et al. found that the combination of 19 habitual or demographic factors (E-score) and 63 CRC-associated SNPs (G-score) had a c-statistic of 0.63 in men and 0.62 in women. Discrimination of this model was significantly better than a model with either the E-score or the G-score alone.

Health economic analyses suggest that further developments within this field are needed for implementation. Thomas et al. conducted a modelling study to estimate the effect of a risk model on expected CRC incidence, mortality, and cost-effectiveness (79). They compared personalized starting ages for FIT screening, based on the risk score, to a fixed starting age for FIT screening. The personalized approach was found to prevent an additional 218 CRC cases and 156 CRC deaths per 100,000 individuals compared to the fixed approach. The highest cost at which the risk assessment could still be cost-effective was £114 per person. However, the c-statistic of the risk model that was used by Thomas et al. was 0.72. This might be optimistic, given that most risk models using SNPs have lower c-statistics when they are externally validated (80). Indeed, a cost-effectiveness study by Naber et al. concluded that changing starting age of screening, stopping age, or personalized screening intervals following polygenic testing would only be cost-effective if the performance of models would improve, the costs of risk-assessment would decrease by at least 30%, or risk-assessment would lead to an increase in screening uptake of at least 5% (81).

## Discussion

At present, CRC screening programs mainly use primary screening colonoscopy or FIT. Most CRC screening programs have applied a one-size-fits-all approach, using uniform start and stop ages, intervals, and cut-offs. Our review sheds light on risk models for screening, which can either be used cross-sectionally by using multiple risk factors or markers to assess current risk, or longitudinally by predicting the risk of future CRC. Although this is only a narrative review of the literature, it is clear that there are many risk models being developed, validated, and considered for future implementation, each having its own advantages and disadvantages (**Table 4**).

**Table 4:** (Potential) advantages and disadvantages of risk models.

	Advantages	Disadvantages
**Cross-section risk models**		
Risk models based on habitual and demographic risk factors	Inexpensive Information available from existing databases	Limited performance Questionnaires may be needed to obtain information
Fecal-based risk models	Markers could be obtained from FIT sample Performance may be better than FIT	Markers may need to be obtained from a whole stool sample Expensive
Blood-based risk models	Some individuals may prefer blood test over stool test Future tests might be able to detect more cancers than CRC	Requires staff and facilities for collection and handling Expensive
**Longitudinal risk models**		
	Enables personalized screening strategies Creates awareness of risk	Limited performance Risk may change when individuals change their lifestyle

Before one of these risk models can be available for screening, several obstacles need to be overcome. First, more evidence of the impact of risk models on actual health outcomes in established screening programs is needed. As indicated by several systematic reviews cited in this paper, most risk models have been studied in case-control studies, in cohort studies of limited size, or in study groups that do not resemble the target population. This may result in an overestimation of the performance of risk models, partly due to a phenomenon called overfitting. Besides, the c-statistic may not be the right performance indicator for risk models intended for screening: it provides information on the performance of a model across all potential cut-off points. In reality, only cut-off points with a substantial positive predictive value for AN (e.g. >20%) may be cost-effective in population-wide screening programs. Therefore, studies that assess the yield and cost-effectiveness of risk models compared to current tests or strategies (e.g. FIT) in head-to-head trials will be essential to determine actual improvements in performance. However, this is easier said than done as such studies require large sample sizes and large budgets. Also, conducting studies within existing settings requires the cooperation of screening organizations, whose primary interest is to maintain continuation of the screening program and not to perform scientific research.

Second, when a risk model is found to have superior performance over current screening tests, the willingness of the target population to undergo screening with this risk model is key. The net benefit of a new superior test may be less than the current test if participation rates are considerably lower. Participation may depend on sample type: data from surveys suggests that screening invitees would prefer a blood-based test over a stool-based test (82–84). However, Zajac et al.’s study consisting of more than 1,500 survey respondents suggests that this effect may be modified by health system interactions: the likelihood of screening participation with a stool-based test was significantly higher when the test could be performed at home compared to a blood-based test in a healthcare setting (85). Another study found that test performance and user-friendliness might be more important attributes than sample type (86). Besides user-friendliness, reimbursement policies for screening tests may influence participation, especially in groups with low socio-economic status. Because the risk of CRC is inversely related to socio-economic status, a decrease of participation to screening in these groups may have a disproportionate effect on the net benefit of a new test (87). Impact studies are needed to assess differences in participation and net benefit of a potential new screening tool.

We discussed multiple cross-sectional risk models which might one day replace FIT. From development studies, it is difficult to appraise whether these risk models will actually be used as a substitute for FIT. Alternatively, risk models may also be used as a tool for triaging or as an add-on test. The latter approach entails that individuals are first screened using an inexpensive test (e.g. FIT) and those tested positive are offered a second, more expensive and accurate test to select who should undergo colonoscopy. Using a risk model as an add-on test could improve a screening program by reducing the number of false positives. Another possibility is that screening programs may offer different screening tests to different risk groups or change the screening interval based on long-term risk of CRC.

Although personalized screening pathways like these and others may improve the yield of screening, it may also increase its complexity. Invitees may not understand why they are offered a different screening method than for example their neighbor or spouse. Also, invitees may not appreciate that a risk model is essentially a mathematical formula and that having a certain risk factor does not mean one is automatically considered risk positive or high risk by the model. For example, an individual who has a family member with CRC might still receive a negative result or low risk from a model that weighs family history of CRC among several other variables. This might lead to confusion, mistrust, and potentially rejection of the result, or lower participation rates. How the results of risk models and its consequences are to be communicated should be addressed and studied before such tools or strategies are implemented.

Ideally, risk models are adaptable to the specific settings and needs of screening programs. In the case of cross-sectional risk models, cut-offs should be easily adjustable to cater for differences in screening capacity or the number of false positives one is willing to accept. To prevent a potential loss of performance, screening programs should also be able to update risk models according to the baseline risk in their target population. Adaptability of risk models can be stimulated by transparent reporting of the model weights and by choosing a model that produces a quantitative output (e.g. a number between 0 and 1).

In this review, we focused only on risk-based screening tools specifically intended for estimating the risk of CRC or AN. However, future risk-based tools might be able to detect several types of cancer. Cohen et al. developed a blood test (CancerSEEK) for 8 types of cancers in a case-control study of 1,005 cancer patients and 812 healthy individuals (88). Although the sensitivity for CRC (close to 70%) was lower than the known sensitivity of FIT, the sensitivity of CancerSEEK for ovarian and liver cancers was more than 95% at a specificity of over 99%. The researchers also developed an algorithm that could predict the most likely anatomic site of the tumor, since a positive test does not indicate where it is located. The algorithm correctly identified the colorectum as most likely tumor site in 84% of CRC patients. It remains to be seen whether such tests can effectively and efficiently reduce cancer-related mortality. Another important subject, but what we believe is beyond the scope of this review, concerns applying quality improvement other than solely the screening test. For example, colonoscopy still has a miss rate for colorectal lesions. The use of artificial intelligence in colonoscopy may decrease the miss rate and consequently improve screening. Also, colonoscopy quality and bowel preparation have substantial impact on an the adenoma detection rate, which affects the risk of interval carcinoma (89, 90). Continuous efforts in improving colonoscopy quality through colonoscopist feedback or auditing for example, may therefore also improve screening (91). Finally, we only briefly discussed studies from upcoming areas in biomarker development, such as VOCs and microbiota, because there is little high-quality evidence to support the benefit of these biomarkers at the moment.

## Conclusions

Although FIT is the current standard for non-invasive CRC screening, it has a low sensitivity for AN. In addition, screening programs mostly use uniform cut-offs, start and stop ages, and screening intervals. Risk models might improve screening by leveraging information from multiple biomarkers and/or risk factors. Screening can be personalized by using risk models to estimate risk of future CRC and by offering different screening strategies accordingly. Providing the evidence in favor for risk models will be difficult as studies require thousands of participants and could take years to complete. Encouragingly, several large trials investigating promising risk models for CRC screening are currently in progress.

## Author Contributions

TK drafted the manuscript. ED and MV reviewed and edited the manuscript. All authors contributed to the article and approved the submitted version.

## Conflict of Interest

ED has endoscopic equipment on loan of FujiFilm and Olympus, and received a research grant from FujiFilm. She also received honorarium for consultancy from FujiFilm, Olympus, GI Supply, CPP-FAP, PAION and Ambu, and speakers’ fees from Olympus, GI Supply, Norgine, IPSEN, PAION and FujiFilm.

The remaining authors declare that the research was conducted in the absence of any commercial or financial relationships that could be construed as a potential conflict of interest.

## Publisher’s Note

All claims expressed in this article are solely those of the authors and do not necessarily represent those of their affiliated organizations, or those of the publisher, the editors and the reviewers. Any product that may be evaluated in this article, or claim that may be made by its manufacturer, is not guaranteed or endorsed by the publisher.
